# Phosphorylation-Assisted Luciferase Complementation Assay Designed to Monitor Kinase Activity and Kinase-Domain-Mediated Protein–Protein Binding

**DOI:** 10.3390/ijms241914854

**Published:** 2023-10-03

**Authors:** Ádám L. Póti, Laura Dénes, Kinga Papp, Csaba Bató, Zoltán Bánóczi, Attila Reményi, Anita Alexa

**Affiliations:** 1 Biomolecular Interactions Research Group, HUN-REN Research Center for Natural Sciences, Institute of Organic Chemistry, 1117 Budapest, Hungary; 2 Doctoral School of Biology, ELTE Eötvös Loránd University, 1117 Budapest, Hungary; 3 Department of Organic Chemistry, Institute of Chemistry, Eötvös Loránd University, 1117 Budapest, Hungary

**Keywords:** cell signaling, protein kinase, luciferase fragment complementation, kinase inhibitors, kinase docking, MAP kinase, RSK

## Abstract

Protein kinases are key regulators of cell signaling and have been important therapeutic targets for three decades. ATP-competitive drugs directly inhibit the activity of kinases but these enzymes work as part of complex protein networks in which protein–protein interactions (often referred to as kinase docking) may govern a more complex activation pattern. Kinase docking is indispensable for many signaling disease-relevant Ser/Thr kinases and it is mediated by a dedicated surface groove on the kinase domain which is distinct from the substrate-binding pocket. Thus, interfering with kinase docking provides an alternative strategy to control kinases. We describe activity sensors developed for p90 ribosomal S6 kinase (RSK) and mitogen-activated protein kinases (MAPKs: ERK, p38, and JNK) whose substrate phosphorylation is known to depend on kinase-docking-groove-mediated protein–protein binding. The in vitro assays were based on fragment complementation of the NanoBit luciferase, which is facilitated upon substrate motif phosphorylation. The new phosphorylation-assisted luciferase complementation (PhALC) sensors are highly selective and the PhALC assay is a useful tool for the quantitative analysis of kinase activity or kinase docking, and even for high-throughput screening of academic compound collections.

## 1. Introduction

Protein kinases control cellular functions via the phosphorylation of downstream substrate proteins and direct mutations in the kinase affecting its enzymatic properties. More indirect systems-level changes in kinase-based signaling networks are known to cause various human diseases [1]. Fortunately, illicit kinase activation could be artificially modulated via ATP-competitive drugs [2]. Mechanistically, this pharmaceutical strategy targets the “druggable” deep nucleotide-binding pocket common to all members of the human kinome (~520 kinases) and there are dozens of specific kinase inhibitors currently used to combat signaling diseases such as cancer or inflammation [3,4]. Unfortunately, acquired resistance to these classical kinase inhibitors, due to somatic mutations or systems-level changes, may render these drugs inefficient in cancer patients [5,6]. This necessitates the development of new inhibitors that mechanistically work differently [2,7,8].

Protein kinases themselves are also regulated by reversible phosphorylation and this relies on protein–protein binding between kinases and their regulators (e.g., upstream kinases, phosphatases, or scaffolds), moreover, efficient phosphorylation of some of the downstream substrates requires additional protein–protein contacts which are distinct from those mediated by the universal substrate-binding pocket located next to the catalytic site/nucleotide-binding pocket. Apart from the enzymatic properties and the capacity to form the catalytic Michaelis–Menten complex, the cellular function and the specific roles of protein kinases are thus also governed by their protein–protein interactions (PPIs). This mechanism is generally referred to as kinase docking and it is common among Ser/Thr kinases [9]. Docking increases the specificity of target site phosphorylation: it involves a dedicated PPI surface on the kinase domain (namely the docking groove) and a linear binding motif sequence from the partner (namely the docking motif) [10]. The docking motif is normally located in a disordered region and contains a set of amino acids fitting into the docking groove upon binding. Docking motifs facilitate the phosphorylation of functionally important phosphorylation target sites by enhanced proximity [11,12]. The binding affinity between the kinase-docking groove and the docking motif is in the low to medium micromolar range [13,14]. In summary, kinase-docking grooves expedite more selective phosphorylation of substrates, while they are also engaged in binding to upstream kinases and/or to phosphatases [15].

Docking interactions have long been known for mitogen-activated protein kinases (MAPKs) and have been discovered more recently for the cAMP-dependent, cGMP-dependent, and protein kinase C (AGC) kinase domain of p90 ribosomal S6 kinase (RSK) [16,17,18]. From a biological standpoint these PPIs help to explain how a certain MAPK or an AGC kinase domain can selectively phosphorylate specific target sites in substrates, since all MAPKs phosphorylate simple S/T-P sites (where P is a proline and serine or threonine will accept the phosphate) and most AGC kinase domains act on similar basophilic R-x-x-S/T target motifs (where R is arginine, x can be any amino acid, and serine or threonine will accept the phosphate) [19]. In summary, interference with kinase docking provides an alternative, possibly more specific, and fundamentally different strategy to ATP-competitive drugs to block kinase function for a specific set of Ser/Thr kinases. There are notable differences between ATP-competitive drugs and docking-interference-based strategies. For example, developing specific protein kinase inhibitors targeting the ATP-binding site may be very challenging because the physiological cellular ATP concentration is very high (mM), requiring nanomolar binding affinity for high potency. Moreover, the catalytic sites of many kinases exhibit remarkable similarity which is a significant challenge in avoiding off-target binding. In contrast to this, the binding partners of kinases are present only in micromolar concentration in the cell and their binding affinities to kinases are also in this far weaker range. Thus, blocking docking-based PPIs may be feasible with weaker binding molecules. However, protein–protein binding surfaces are generally shallow compared to the deep ATP-binding pocket which could hamper the identification of good binders [20,21].

MAPKs and their substrates play pivotal roles in various human diseases and this kinase group includes the extracellular signal-regulated kinases (ERK), c-Jun N-terminal kinases (JNK), and p38 kinases that regulate diverse physiological processes such as cell proliferation, differentiation, apoptosis, and inflammation [22,23,24]. ERK and RSK are key downstream constituents of the epidermal growth factor (EGF)/Ras/Raf/MEK/ERK signaling pathway. Structurally, RSK is composed of two kinase domains tethered by a flexible linker [25]. The N-terminal AGC-type kinase domain (NTK) catalyzes downstream substrate phosphorylation following its co-activation by phosphoinositide-dependent kinase 1 (PDK1) and its C-terminal kinase domain (CTK; calcium/calmodulin-dependent kinase (CAMK) domain). ERK—via its docking groove—binds to RSK’s C-terminal tail harboring a MAPK-binding linear D(ocking)-motif, which then turns on the phosphorylation cascade leading to RSK substrate phosphorylation [26]. Interestingly, this latter may also depend on kinase docking where the RSK NTK binds to so-called DxVF motifs, henceforth referred to as VF-motifs, found in RSK substrates [18]. Furthermore, MAPKs in addition to their first-discovered D-groove, binding to D-motifs, also contain an F-groove, unrelated to the former, binding to so-called FxFP motifs from substrates [27,28,29]. In summary, the MAPKs and RSK NTK provide an excellent system to test the feasibility of kinase docking-interference-based strategies. Well-established ATP-competitive inhibitors exist for MAPKs (ERK1/2, JNK1/2/3, and p38α/β) as well as for RSK NTK, but PPI-blocking drugs for these enzymes are just emerging and this strategy is, by far, less-explored [30,31,32,33]. We posit that new biochemical tools/assays will likely facilitate the discovery of novel compounds targeting the docking grooves of these kinases.

In the present study we report the development of an in vitro kinase assay based on luciferase fragment complementation and specific phospho-amino-acid-binding domains. We show that the phosphorylation-assisted luciferase complementation (PhALC) concept could be used in academic labs for the screening of compound libraries to identify small compounds that block kinase function. The PhALC assay uses self-made reagents, cutting down on screening costs, and is particularly geared towards finding compounds that interfere with kinase docking. The assay was validated against known ATP-competitive drugs and allows rapid quantitative assessment of inhibitor efficiency (IC_50_). More importantly, the assay was also validated with MAPK D- or F-groove and RSK NTK docking groove binding molecules and it is suitable to identify compounds with interesting docking-interference patterns based on a pilot study with a small academic compound collection.

## 2. Results

### 2.1. Concept of the PhALC Assay—RSK Sensor

In natural phosphorylation-based signaling networks protein kinases, as the “writers”, phosphorylate substrates at target motifs located in biologically critical protein regions [34]. Phospho-amino acids may then be bound by dedicated phospho-amino-acid-binding domains, as “readers”, which have naturally evolved to recognize specific phosphorylation events in the cell [35,36]. Target motif sequences therefore have not only evolved for optimal modification by the “writers” but they are also compatible with the “readers”. This inspired the design of a new kinase assay concept in which natural recognition domains work together with selected target-motif-containing sensors to monitor the phosphorylation of the latter as substrates by selected kinases.

In most in vitro kinase assays, protein phosphorylation is monitored by the incorporation of radioactive phosphate into the substrate, by mass spectrometry, or phosphorylation, and is recognized by artificial phospho-amino-acid-specific antibodies (Western blotting), or monitored indirectly through nucleotide cofactor hydrolysis (ADP-Glo kinase assay). These assays do not harness the natural biological specificity of intracellular phospho-amino-acid-recognizing domains and they require expensive reagents (e.g., phospho-specific antibodies or radioactive reagents) or instruments (e.g., scintillation counters and radioactive facilities or high-end mass spectrometers) that may not be available for most molecular biology labs.

**Figure 1:**
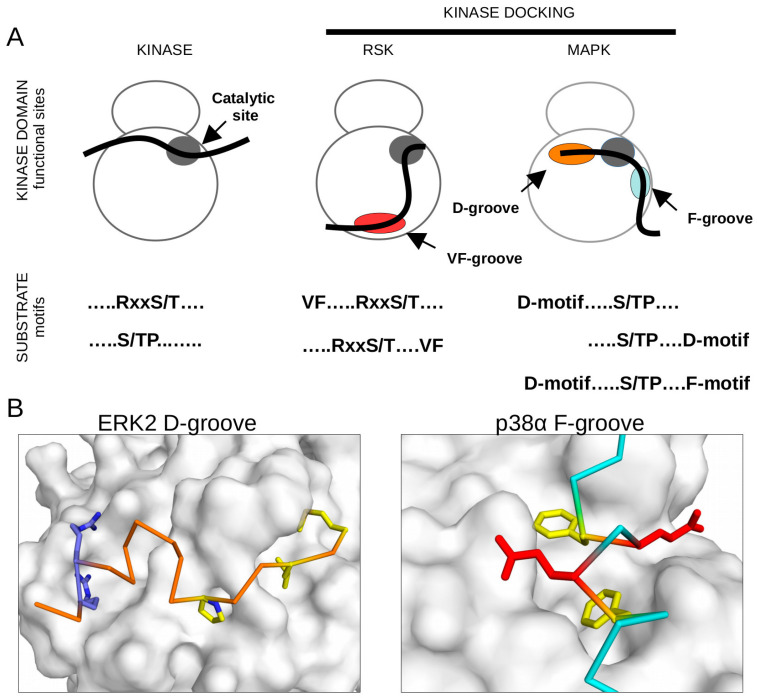
Kinase-docking-dependent substrate phosphorylation. (**A**) Panels show schemes of target-motif phosphorylation, an RxxS/T motif for AGC kinase and S/TP motif for MAPKs in substrates (see solid black line binding to the substrate-binding pocket next to the catalytic site shown in dark gray), VF-groove-assisted phosphorylation of AGC kinase target sites for RSK, and the involvement of the D-groove or the F-groove for MAPKs. (**B**) Panels show the MAPK D-groove or the F-groove from the crystal structures of the ERK2-pepMNK1 or p38α-ATF2(pepFENEF) complexes (PDB IDs: 2Y9Q and 6ZQS, respectively). The MAPK structure is shown in surface representation, the docking peptide backbone is colored orange or cyan, and side-chains show key hydrophobic (yellow) or charged residues (blue: Arg/Lys, red: Glu/Asp) fitting into the distinct MAPK-docking grooves.

Currently, we know of dozens of different phospho-amino-acid-binding domains (e.g., Src homology domain 2 (SH2) or phosphotyrosine-binding (PTB) domain for phosphotyrosine and 14-3-3 or WW for phosphoserine/-threonine). These are known to bind to specific phospho-amino-acid-containing motifs with increased affinity, which—as a heterologous interaction—may be indirectly exploited to monitor protein phosphorylation via luciferase enzyme complementation. Luminescence measurements are sensitive, have great signal-to-noise properties, and can be measured in a standard luminometer in microplates. The phosphorylation-assisted luciferase complementation (PhALC) assay requires two protein constructs: the SENSOR, which will be modified at specific sites by the examined kinase, and the RECOGNITION CONSTRUCT (RC), which will “read” the phosphorylation of the sensor by triggering the complementation of the luciferase (NanoBiT, whose two fragments, SmBiT and LgBiT, are located on the sensor or on the RC, respectively). The NanoBiT luciferase enzyme is particularly suited for PhALC, since it was originally developed as a dynamic protein–protein-interaction-monitoring tool with very low spontaneous assembly between its two fragments [37]. The RC may contain any phospho-target-motif-binding domain whose binding specificity matches to the target motif preference of the investigated kinase. Since the constructs are modular, the sensor could be designed to contain a docking motif in addition to its target motif. Such chimera constructs, for which efficient phosphorylation of the target motif will depend on kinase docking, could be used to characterize a docking motif or kinase-docking-groove-mediated binding (Figure 1A,B).

**Figure 2:**
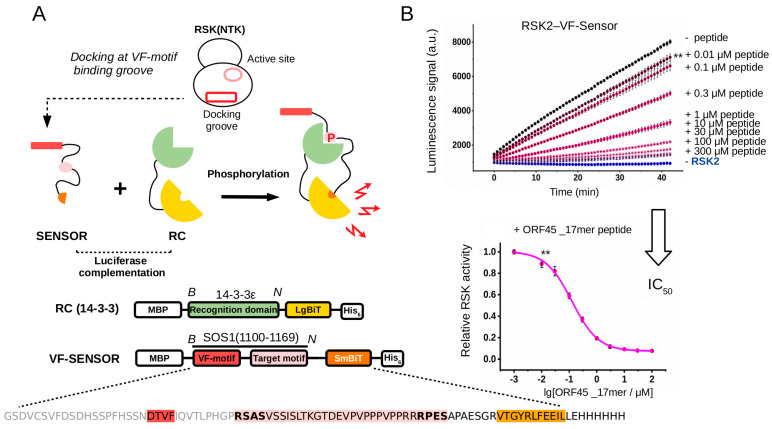
Principle of the PhALC assay and its validation on RSK-docking-motif-mediated binding. (**A**) The sensor construct contains the SOS1(1100–1166) region that harbors the VF-motifs and the RxxS/T target sites (VF-Sensor). The latter is bound by the recognition construct (RC) upon phosphorylation. The RC harbors a phosphoserine/-threonine-binding 14-3-3ε domain and the phosphorylation-dependent interaction between the VF-Sensor and RC will facilitate the formation of active luciferase. (The VF-Sensor and the RC are tagged with SmBit and LgBiT fragments of the NanoBiT luciferase, respectively, and also contain N- and C-terminal affinity tags for fast purification; MBP—maltose binding protein, His_6_—hexahistidine tag). The VF-Sensor and the RC were used in 1 μM and 0.5 μM concentrations, full-length active RSK2 was used in 20 nM concentration and the reaction was started by the addition of ATP. (**B**) Panels show that the kinetic rate of the luminescence signal correlated with the degree of VF-Sensor phosphorylation by RSK. A VF-motif-containing peptide added in trans competed for binding with the VF-Sensor and thus decreased the rate of the luminescence signal (upper panel). RSK activity was compared to the rate of the reaction containing no peptide (−peptide; the panel shows the raw kinetic luminescence signal; a.u. artificial units; −RSK2: no kinase added). The IC_50_ value of peptides can be determined when relative RSK activity is plotted as the function of peptide concentration added in trans (lower panel showing the results obtained with ORF45_17mer peptide). Error bars show SD based on three measurements. (*B* and *N* denote BamHI and NotI cloning sites with which the functional elements of the sensor and the RC could be modified in the DNA expression plasmids.) Asterisks, here shown only for the lowest concentration of the competitor peptide compared to “−peptide”, indicate results of a two-sided, unpaired *t*-test (**: *p* < 0.01).

First, we applied the PhALC concept on RSK (Figure 2A). The RSK Sensor is a fusion between one of the two fragments of the split luciferase and the ~70-amino-acids-long SOS1 fragment, including the VF-motifs and target sites. SOS1 is a natural substrate of RSK phosphorylated by activated NTK at two specific RxxS/T target motifs [38]. The phosphorylated sensor can bind to the recognition domain containing chimera which is a fusion construct between the other fragment of the luciferase and the phosphopeptide-binding 14-3-3ε domain known to bind phosphorylated SOS1 [39]. VF-motif-based docking to the sensor, its phosphorylation by activated RSK, recognition-domain recruitment, and, ultimately, luciferase complementation increased the luminescence signal that was measured in a luminometer using microplates. Next, we tested if kinase docking could be quantitatively analyzed in the newly developed RSK PhALC assay (Figure 2B). A known docking motif of the RSK NTK, derived from the Kaposi’s-sarcoma-associated herpesvirus (KSHV) ORF45 protein, was added in trans, in increasing amounts, and the initial kinetic slope of the luminescence signal was measured. These measurements showed that the ORF45 peptide interfered with kinase-docking-facilitated phosphorylation and the IC_50_ value for the 17-amino-acids-long peptide containing the core VF-motif was found to be in the low micromolar range, as expected [18].

### 2.2. MAPK-Docking-Based Tests

**Figure 3:**
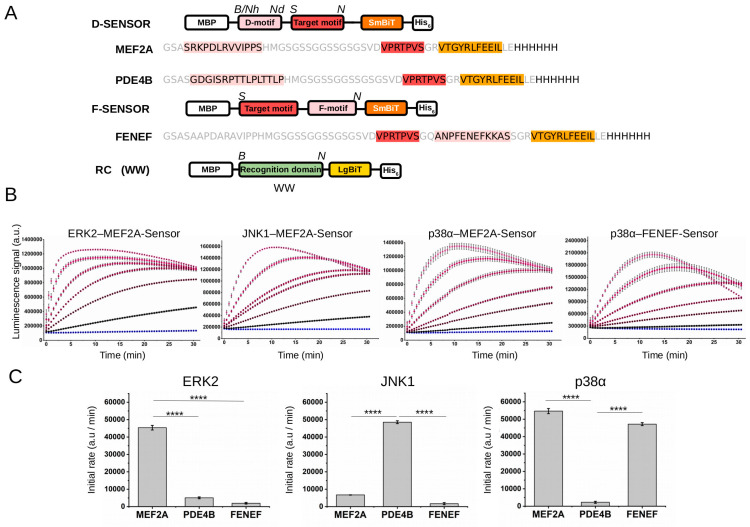
MAPK-docking-responsive PhALC. (**A**) The schematic of the MAPK-Sensors: D-Sensors contain the SmBiT part of the NanoBiT luciferase and either the MEF2A or the PDE4B docking motifs located N-terminal to the target motif, while the F-Sensor contains the FENEF docking motif C-terminal to the same target motif. The same recognition construct (RC), comprised of a phosphothreonine-binding WW domain from Pin1 and the LgBiT part of NanoBiT, could be used to bind to any of the phosphorylated sensors above. All constructs contain an N-terminal MBP- and His_6_-tag for easy purification of recombinant expressed proteins. (*B*, *Nh*, *Nd*, *S*, and *N* denote BamHI, NheI, NdeI, SalI, and NotI restriction enzyme cleavage sites that can be used to modify DNA expression plasmids of the shown sensor or RCs.) (**B**) The PhALC signal depends on the concentration of the activated MAPKs. Double-phosphorylated, active MAPKs were added in increasing amounts into the reaction mix: no enzyme (blue) or 0.1 nM, 0.5 nM, 1 nM, 2 nM, 5 nM, or 10 nM (magenta). The luminescence signal was monitored for up to 30 min after the addition of ATP. (**C**) Comparative analysis of the three sensors (MEF2A-Sensor, PDE4B-Sensor, and FENEF-Sensor) with ERK2, JNK1, and p38α. Panels show the initial rate of luminescence from the linear range of signal (~up to 10 min) with the different sensors determined under the same conditions as on panel B but using 0.3 nM active pp-ERK2, 1 nM pp-JNK1, or 1 nM pp-p38α. Error bars show SD based on three measurements. Asterisks indicate results of a two-sided, unpaired *t*-test (****: *p* < 0.0001).

Similar to what was described earlier with the RSK Sensor, to be able to monitor kinase docking for MAPKs, the NanoBit luciferase fragment complementation-based PPI assay concept was modified so that MAPK-docking-mediated phosphorylation of an artificial substrate sensor could also be monitored in a microplate reader. Briefly, a general MAPK phosphorylation target motif (S/TP) was positioned C-terminal from an MAPK-binding D-motif, or N-terminal from an F-motif, and this SENSOR construct was fused with the small fragment of the luciferase enzyme. In the recognition construct (RC), a WW domain binding specifically to the phosphorylated MAPK target motif was fused with the large fragment of the luciferase [40]. We made two different D-Sensors containing different D-motifs: a short MEF2A D-motif-containing peptide was earlier shown to bind both to ERK2 and p38α, while the PDE4B motif binds only to JNKs (MEF2A-Sensor and PDE4B-Sensor) [13,15]. In addition, an F-groove binding short peptide from ATF2 known to specifically bind to phosphorylated p38α was used in the F-Sensor (henceforth referred to as the FENEF-Sensor) [29] (Figure 3A).

Next, we addressed how activated MAPK concentration affects the luminescence signal for ERK2–MEF2A-Sensor, JNK1–PDE4B-Sensor, p38α–MEF2A-Sensor, and p38α–FENEF-Sensor pairs. This showed that, as expected, the increase in the rate of the luminescence signal correlated with the amount of the active enzyme, and, more importantly, 1 nM or even subnanomolar amounts of enzyme gave robust signal with 1 μM sensor and RC (Figure 3B). Moreover, a comparative analysis showed, as expected, that ERK2 and JNK1 phosphorylate only the MEF2A- or the PDE4B-Sensor, respectively, while p38α can phosphorylate both the MEF2A- and the FENEF-Sensor (since both docking motifs from these two sensors are known to bind this MAPK, albeit via two distinct docking grooves, the D- or the F-groove, respectively) (Figure 3C).

### 2.3. Measurements with Known ATP-Competitive Inhibitors and Docking-Interfering Peptides

**Figure 4:**
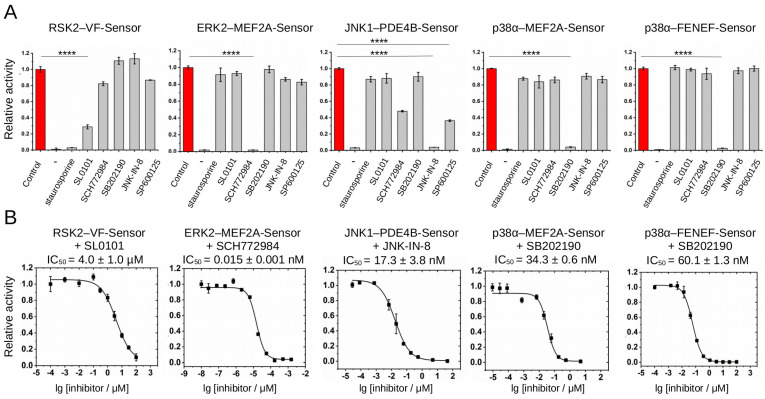
Validation of different PhALC assays with known ATP-competitive inhibitors. (**A**) Summary of PhALC assay results showing the effect of known ATP-competitive inhibitors. Panels show the effect of inhibitors (10 μM) on five different PhALC assays. The initial luminescence rate was normalized to the control reaction that contained no inhibitor (control), while the negative control reaction contained no enzyme (−). (**B**) Summary of PhALC IC_50_ measurements using RSK inhibitor (SL0101), ERK inhibitor (SCH772984), JNK inhibitor (JNK-IN-8), and p38 inhibitor (SB202190) in respective PhALC assays. (Note that the range of the X axis is adjusted to inhibitor strength.) Asterisks, here shown for the cognate MAPK inhibitors compared to “Control”, indicate results of a two-sided, unpaired *t*-test (****: *p* < 0.0001).

Apart from kinase docking, the PhALC assay may also be used as a general kinase-activity reporter system. To this end, we tested the effect of six different ATP-competitive inhibitors: staurosporin, SL0101 (RSK NTK inhibitor), SCH772984 (ERK inhibitor), SB202190 (p38 inhibitor), JNK-IN-8 (a highly selective JNK inhibitor), and SP600125 (another widely used JNK inhibitor) in 10 μM concentration in all five newly developed PhALC assays (RSK2–VF-Sensor, ERK2–MEF2A-Sensor, JNK1–PDE4B-Sensor, p38α–MEF2A-Sensor, and p38α–FENEF-Sensor) [41,42,43,44,45] (Figure 4A). This showed that staurosporin efficiently blocked RSK, but the activity of MAPKs was unaffected with this inhibitor and SCH772984 and SB202190 blocked ERK2 and p38α, respectively, while both JNK inhibitors had the strongest effect on JNK1. This matches the expected inhibitory profile of the tested drugs. Next, we used the PhALC assay and determined the in vitro IC_50_ of SL0101 on RSK, SCH772984 on ERK2, and JNK-IN-8 on JNK1, which all gave values matching those that had been determined earlier for these kinases by other methods: RSK2/SL0101, 4 μM; ERK2/SCH772984, 0.015 nM; and JNK1/JNK-IN-8, 17 nM [41,42,44]. Moreover, for the p38-specific inhibitor (SB202109) assays with both the MEF2A-Sensor or the FENEF-Sensor gave similar values (34 and 60 nM, respectively), as expected (Figure 4B).

**Figure 5:**
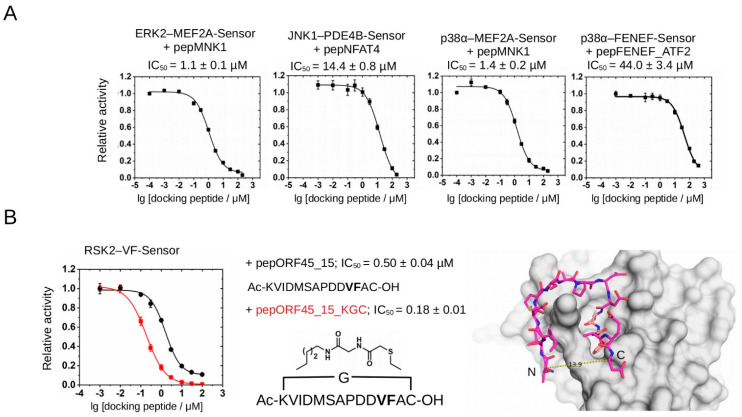
Validation of different PhALC assays with kinase-docking-blocking peptides. (**A**) Summary of PhALC IC_50_ measurements with known D-motif peptides (pepMNK1 or pepNFAT4) or F-motif peptide (pepFENEF_ATF2). (**B**) Comparison of RSK2–VF-Sensor IC_50_ measurements with a linear (pepORF45_15; black) or a cyclic ORF45 (pepORF45_15_KGC; red) peptide. “Ac” and “OH” denote acetylated N-terminus and free carboxyl group at the C-terminus of the peptides, respectively. The chemical formula shows the structure of the artificial linker, with a glycine (G) inserted between the side chains of the N-terminal lysine and the C-terminal cysteine, thus making a circular peptide. The panel on the right highlights the close proximity of the N- and C-termini of a 15mer from the bound ORF45 peptide from the crystal structure of the RSK2(NTK)-pepORF45 crystallographic complex (PDB ID: 7OPO). This panel also shows how the VF-motif (colored in salmon) binds into a small hydrophobic pocket on the RSK(NTK). Note that in the ORF45_15 peptide the original Thr and Glu amino acids at the N- and C-termini were replaced with Lys and Cys to able to link them (~14 Å) via their side chains covalently (pepORF45_15_KGC). Error bars show SD based on three measurements.

Finally, we validated all five assays by using chemically synthesized D-, F-, or VF-motif containing peptides, which were added in trans (Figure 5A). These experiments showed that IC_50_ values determined this way showed an excellent agreement with the binding affinity of docking peptides or with other independent measurements: ERK2/pepMNK1 PhALC(IC_50_) = 1.1 μM vs. 1.2 μM binding affinity of pepMNK1 to ERK2; JNK1/pepNFAT4 PhALC(IC_50_) = 14.4 μM vs. 7.1 μM binding affinity of pepNFAT4 to JNK1; p38α/pepMNK1: PhALC(IC_50_) = 1.4 μM vs. 0.4 μM binding affinity of pepMNK1 to p38α [13]; p38α/FENEF PhALC(IC_50_) = 44 μM vs. ~30 μM IC_50_ determined earlier in a Western-blot-based assay [29]; and RSK2/ORF45 _15 PhALC(IC_50_) = 0.5 μM vs. 2 μM binding affinity for an ORF45 peptide similar in length binding to RSK NTK [18]. Moreover, a newly synthesized cyclic version of the ORF45 VF-motif peptide displayed a close to three-fold lower IC_50_ value compared to the linear version, suggesting that cyclization may create a more rigid structure well-suited to adopting an optimal binding conformation in the docking groove—as the artificial covalent link between the N- and C-terminal amino acids had been designed based on the crystal structure of the RSK(NTK)-pepORF45 crystallographic complex [18] (Figure 5B).

### 2.4. PhALC in Human-Cell Lysates

**Figure 6:**
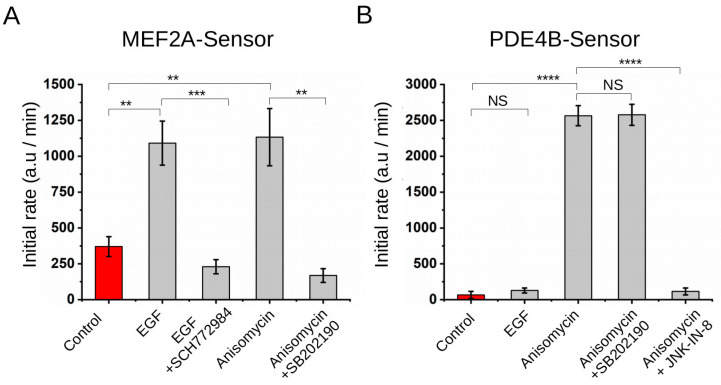
Application of the PhALC assay in HEK293T cell lysates. Summary of PhALC measurements with (**A**) MEF2A-Sensor and (**B**) PDE4B-Sensor using EGF-treated or anisomycin-treated HEK293 cells. HEK293T cells were pre-incubated with no inhibitor (control in red) or with 10 μM inhibitor before EGF (100 ng/mL) or anisomycin (10 μg/mL) stimulation in 12-well plates. Cells were lysed and some of the lysate was added into the MEF2A-Sensor or PDE4B-Sensor containing standard PhALC assay reaction mix. The panels show the initial rate of luminescence. Note that EGF turns on ERK activity while anisomycin turns on p38 and JNK. (SCH772984 is an ERK-specific, SB202190 is a p38-specific, and JNK-IN-8 is a JNK-specific inhibitor.) Error bars show SD based on three measurements. Asterisks indicate results of a two-sided, unpaired *t*-test (NS: nonsignificant, **: *p* < 0.01, ***: *p* < 0.001, ****: *p* < 0.0001).

In the previous in vitro tests MAPKs were added as purified active enzymes. We tested whether the developed sensor/RC pairs could also be used to report on MAPK activity levels of human cell lysates. HEK293T cells were treated with epidermal growth factor (EGF; 100 ng/mL) or anisomycin (10 μg/mL) which are known to trigger ERK or stress MAPK (p38/JNK) activation, respectively, and 5 μL of these cell lysates containing a total amount of 6 μg of proteins was added into different in vitro PhALC tests. We found that cell lysates formerly treated with EGF contained more ERK activity because the MEF2A-Sensor gave a higher PhALC signal compared to control cell lysate and, as expected, the JNK-specific PDE4B-Sensor system stayed unaffected. Moreover, the ERK-specific PhALC signal could be blocked by the ERK inhibitor (SCH772984), while the positive effect of anisomycin treatment on p38/JNK activation was blocked by p38-specific (SB202190) or JNK-specific (JNK-IN-8) inhibitors (Figure 6A,B). These results show that the assay could also be used to monitor the specific kinase activity of human cell lysates.

### 2.5. Compound-Collection Screening with Parallel PhALC Assays

The developed PhALC tests required low amounts of activated enzymes and only low micromolar sensor/RC concentration, moreover, the initial slope of luminescence upon addition of ATP provided a robust read-out modality with good signal-to-noise ratio which was well-suited to monitor kinase activity in vitro. We posited that the PhALC assay is a good biochemical tool to search for novel compounds affecting RSK or MAPK activity. To this end, we have assembled an academic in-house compound collection comprised of ~500 molecules, and 88 unique compounds from this collection, representing a chemically diverse set, were tested in RSK2–VF-Sensor, ERK2–MEF2A-Sensor, p38α–MEF2A-Sensor, p38α–FENEF-Sensor, and JNK1–PDE4B-Sensor assays (Supplementary Figure S1). The Z-scores of the initial luminescence measurements in these tests were found to be 0.8–09, indicating that these assays are indeed suitable for multiple parallel tests. Screening results with these five different tests showed that several compounds (in 200 μM concentration) greatly decreased the initial slope of the luminescence signal.

**Figure 7:**
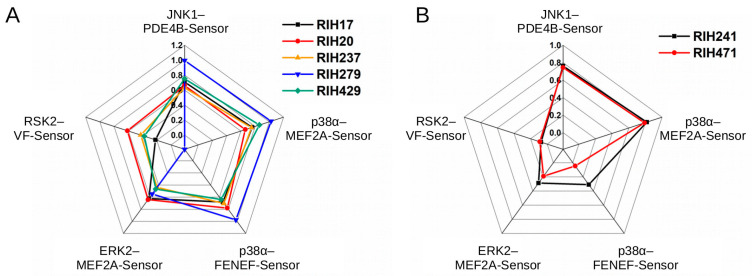
Summary of screening results with an academic compound collection. Inhibitory specificity profile of compounds that decreased the initial luminescence rate more than 70% in any of the five PhALC assays in the primary screens (see Supplementary Figure S1). (**A**) Panel shows this inhibitory profile for promiscuous compounds, where the axes show the degree of inhibition (0: no inhibition, 1:100% inhibition). (**B**) Panel shows the inhibitory profile of two, more selective compounds (RIH241 and RIH471).

Since the tested compound set was chemically highly diverse, the “hit” molecules may lower the luminescence signal due to mechanistically different reasons: they may interfere with kinase docking or kinase enzymatic activity, which would make them indeed potentially interesting, however, they may also be “artifacts” if they, for example, block the complementation of luciferase or interfere with the binding of RC to the phosphorylated sensor. Briefly, since the PhALC assay is complex, it is prone to identifying a great number of false positives. Notwithstanding to this, the interesting candidates could be selected if the PhALC tests are performed on the same set of compounds in parallel, because compounds blocking the luminescence signal in all tests are likely not interesting, since they are neither specific to any kinase nor likely suited to block kinase docking in particular. In contrast, compounds specifically affecting the signal only in specific test(s) are promising candidates. Seven of the 88 tested compounds caused greater than 70% decrease in the luminescence signal in any of the five PhALC tests. However, five of these compounds affected the signal in a fairly unspecific manner in all tests, rendering them to be false hits. More importantly, two compounds (RIH241 and RIH471) selectively blocked the signal in the stress-activated MAPK/D-motif-Sensor tests (p38α–MEF2A-Sensor and JNK1–PDE4B-Sensor) (Figure 7A,B).

## 3. Discussion

Antibody- or mass-spectrometry-based standard approaches require expensive reagents or instrumentation to interrogate proxies for kinase activity. Moreover, these are not well-suited for comparative and quantitative kinetic analysis of parallel kinases. The power of fluorescence-based kinetic analysis for this group of enzymes was demonstrated using a phosphorylation-sensitive artificial amino acid, Sox, coupled with kinase-selective substrates, and this technology was used to monitor the activity of several Ser/Thr kinases as purified proteins, from cell lysates or in unfractionated, homogenized biological samples/tissues [46,47,48]. Phosphorylation of a kinase target site is read out in a “chemical” manner, via phosphorylation-induced chelation of a Mg^2+^ which changes the fluorescence properties of the artificial amino acid coupled next to the kinase’s target Ser/Thr residue. The specificity of such Sox peptides could be greatly enhanced by using docking motifs for MAPKs [49,50,51]. Notably, FRET-based sensors incorporating genetically encodable fluorescent probes can also be used to monitor kinase activity, however these probes often only produce a modest change in the fluorescence signal upon phosphorylation [52].

According to our knowledge, luminescence—which is a more robust, easily measurable biophysical signal—has not been widely used to examine kinases. In this study we reported the design and validation of different sensor and RCs that can be used to monitor the activity of kinases from two different Ser/Thr kinase groups (RSK and MAPKs) by measuring the luminescence signal of simple biochemical reactions or more complex cell lysate samples. Phosphorylation of a kinase target site is read out in a “biological” manner, via using natural phospho-amino-acid-binding domains that can selectively recognize the phosphorylated form of the kinase’s target Ser/Thr residue. Similar PhALC assays, namely new sensor/RC pairs, could likely be developed for other kinases. In principle, any kinase phosphorylating a target motif in the SENSOR binding to a natural phospho-amino-acid-binding domain in the RC could be monitored. Moreover, by exploiting the natural specificity of target motif sequences and/or kinase docking motifs, the PhALC signal could be made specific to certain kinases. The PhALC concept, for example, could likely be easily adopted to also monitor tyrosine kinase activity, since SH2 domains have naturally evolved to bind to specific phosphotyrosine target motifs [53].

The modular design of the SENSOR construct and the fact that these could be independently combined with different RECOGNITION constructs provide great flexibility for different applications and we foresee that PhALC assays will be useful tools to (1) monitor kinase activity to characterize inhibitors, (2) test sequence determinants important for kinase docking and specificity, and (3) test academic compound collections to identify modulators of kinase function (activity vs. protein–protein interactions). The caveat for the latter is that in the PhALC assay, apart from the phosphorylation of the sensor, the luminescence signal requires additional protein–protein binding events, such as the binding of RC to sensor as well as the complementation of the luciferase. Our study with an academic compound collection showed that the false-positive rate may indeed be high due to the complexity of the molecular events leading to the final signal, but more importantly, this could be mitigated by carrying out parallel assays with different kinases on the same set of small molecules.

Two other commonly used screening methods for PPI detection that are also useful for kinase drug discovery are TR-FRET and AlphaScreen [54,55]. Time-resolved fluorescence energy transfer (TR-FRET) is the combination of time-resolved fluorometry (TRF) with Förster resonance energy transfer (FRET). Homogeneous TRF (HTRF) is a no-wash technology utilizing rare-earth lanthanides with long-emission half-lives as donor fluorophores and combines standard FRET with time-resolved fluorescence measurement. AlphaScreen is a non-radioactive amplified luminescent proximity homogeneous assay based on using functionalized beads. When biological interaction brings the beads close, the signal is greatly amplified because the laser excitation of the “Donor” bead generates singlet oxygen diffusing to the “Acceptor” bead triggering the emission of light. The drawback of these methods is that they require high-end microplate readers equipped with lasers and sensitive optical detection units for high-throughput screening as well as expensive reagents (antibodies, functionalized beads, etc.) that can be obtained only from commercial vendors.

The PhALC assay measurements on the tested kinases and inhibitors gave IC_50_ values that matched those determined with other standard methods. According to our experiments, spontaneous luminescence of compounds in the PhALC reaction buffer, which could potentially interfere with the luciferase-generated signal is not a pragmatic problem, as opposed to fluorescence-based readouts where auto-fluorescence of compounds is often limiting. A pragmatic advantage of the PhALC assay over other kinase assay methods is that the key reagents could be self-produced using bacterial expression plasmids and these could be easily modified and tailored. Apart from the luciferase substrate (coelenterazine), assay components can be produced in a standard molecular biology lab, and the assay can be carried out in different microplate formats requires low amounts of active kinase due to its high sensitivity. Moreover, we showed that the assay can be used not only with purified kinases but the active enzyme could be produced in human cell lines by a natural activation scheme if recombinant active kinase production is limiting, and then the cell lysate can be directly added into the PhALC assay reaction mix.

## 4. Materials and Methods

### 4.1. Design and Cloning of the PhALC Sensors

A modified pET15 vector (Novagen) including N-terminal MBP-tag and C-terminal hexa-histidine tag was used to generate the expression vectors of the different SENSORs and RCs. The VF-Sensor includes the human SOS1 DNA-sequence (1100-1166; Uniprot: Q07889) and was generated by annealing oligos. 14-3-3ε-LgBiT RC includes human full-length 14-3-3ε (Uniprot: P62258) and was cloned from HEKT cDNA by PCR and restriction cloning. MEF2A-Sensor, FENEF-Sensor, PDE4B-Sensor, and WW-LgBiT RC for the MAPK system were cloned by annealing oligos. The modular structure of the insert makes it possible to change the sequence of the motifs using different restriction-enzyme cleavage sites. In the case of the MAPK Sensors, the D-motif can be changed by BamHI/NheI-NdeI, the target motif by SalI-NotI, and the target motif + F-motif box by SalI-NotI restriction enzymes. The sequence of the RSK Sensor (VF-motif + target motif box) can be changed by BamHI-NotI restriction enzymes. In the RCs the phospho-amino-acid-binding domain can be changed by BamHI-NotI.

### 4.2. Protein Expression and Purification

SENSOR and RC proteins were expressed in BL21 (DE3) cells in LB medium containing 0.1 mg/mL ampicillin, overnight at 18 °C. Expression was induced with 200 µM IPTG at 0.8 OD. Cells were harvested with centrifugation (5000 rpm, 10 min, 4 °C) and resuspended in lysis buffer (50 mM sodium phosphate, pH = 8.0; 300 mM NaCl; 0.5 mM PMSF; 2 mM benzamidine; 2 mM β-mercapto-ethanol; 0.1% Igepal; and protease-inhibitor tablet (SigmaFast protease inhibitor, S8830)). Cells were lysed by freezing and sonication in the presence of 10 µg/mL DNase. Proteins were separated by nickel-affinity chromatography (GE Healthcare Ni Sepharose 6 Fast Flow #17531803). The column was washed with washing buffer (50 mM disodium phosphate, pH = 8.0; 300 mM NaCl; and 20 mM imidazole) and eluted with elution buffer (20 mM Tris, pH = 8.0; 200 mM NaCl; 400 mM imidazole; 10% glycerol; 2 mM β-mercaptoethanol; and 0.1% Igepal). The proteins 14-3-3-LgBiT RC, VF-Sensor, and WW-LgBiT RC were further purified on maltose resin (Abcam, Cambridge, UK, ab270538) using 20 mM Tris, pH = 8.0; 300 mM NaCl buffer containing 0.1% Igepal; 2 mM β-mercaptoethanol for washing; and an additional 30 µM maltose added for elution. The proteins MEF2A-Sensor, FENEF-Sensor, and PDE4B-Sensor were further purified by HiTrap Q FF cation-exchange column (Cytiva, Marlborough, MA, USA, #17505301), and linear gradient of buffer A (20 mM Tris, pH = 8.0; 50 mM NaCl; and 10% glycerol) and buffer B (20 mM Tris, pH = 8.0; 1 M NaCl; and 10% glycerol) was applied for the elution. The purified proteins were aliquoted at 20–100 µM concentration and stored at −80 °C. Activated full-length RSK2, used with the VF-Sensor, was expressed in SF9 cells using pFastBacHTb vector, which contains an N-terminal 6xHis-tag [18]. The SF9 cell lysate was subjected to Ni-NTA purification, and the eluted sample was aliquoted and stored at −80 °C. Active, phosphorylated ERK2, p38α, and JNK1 proteins were produced in Rosetta2 (DE3) *E. coli* cells by co-expressing them with constitutively active GST-tagged MAP2Ks using bicistronic vectors [15]. All the purified protein samples contained 2 mM TCEP and 10% glycerol and were aliquoted, flash-frozen in liquid nitrogen, and stored at −80 °C. The purity of the proteins was analyzed by SDS-PAGE.

### 4.3. Cyclization of the ORF45 Peptide

Linear peptides were ordered from NovoPro Bioscience Inc. in the form of acetate salt and their purity were above 95%, tested by HPLC. The cyclic peptide was synthesized manually using Fmoc/tBu solid-phase peptide synthesis (SPPS) on Rink amide MBHA resin [56]. In the last step, the chloroacetyl group was introduced using pentachlorophenyl chloroacetate. The cleavage of the peptide was accomplished using 5 mL TFA containing 0.365 g phenol, 0.25 mL distilled water, 0.25 mL thioanisole, and 0.125 mL TIS as scavengers. The cleaved peptide was precipitated and washed twice by dry diethyl ether, dissolved in 10% acetic acid, lyophilized, and purified by semi-preparative HPLC. The peptide was cyclized in tris(hydroxymethyl)aminomethane (Tris) buffer for a day at room temperature. The crude product was purified by semi-preparative HPLC. The chemical characterization of the purified compounds was done by analytical RP-HPLC and ESI-MS (ORF45_15: Ac-KVIDMSAPDDVFAEC, R_t_: 12.4 min, M_calc_: 1719.8, M_meas_: 1719.5; ORF45_15_KGC: Ac-K(G)VIDMSAPDDVFAEC, R_t_: 12.3 min, M_calc_: 1776.8, M_meas_: 1776.6).

### 4.4. Kinase Assays with HEK293T Cell Lysates

HEK-293T (ATCC, CRL-3216) cells were cultured in Dulbecco’s modified Eagle’s medium (DMEM, Gibco, Billings, MN, USA) supplemented with 10% fetal bovine serum (FBS) and gentamicin (50 μg/mL) at 37 °C in an atmosphere of 5% CO_2_. Confluent cells from 12-well plates were incubated with inhibitor for 2 h and then the cells were stimulated with EGF (100 ng/mL, Sigma-Aldrich, St. Louis, MO, USA, #E9644) for 10 min or anisomycin (10 µg/mL, Sigma, #A9789) for 20 min. After stimulation, the cells were washed twice with ice-cold PBS and collected by cold lysis buffer (50 mM Tris, pH = 7.4; 150 mM NaCl; 1% TritonX-100; 1 mM EGTA; 2 mM DTT; 2 mM benzamidin; SigmaFast protease inhibitor tablets; and PhosStop phosphatase inhibitor, Roche, Basel, Switzerland, # 04906837) and were frozen and thawed several times. Cells from one well of the 12-well plate were lysed in 150 µL buffer, sonicated for 5 sec with a Branson Digital Sonifier (Amplitude 30%), and then centrifuged at 20,000 *g* at 4 °C for 20 min. Then, 5 µL of cell lysate was used for the PhALC assays in 25 µL reaction volume in a white 384-well microplate.

### 4.5. PhALC Assay Measurements and IC_50_ Determination

In the case of the RSK-PhALC assay the reaction mixture contained 0.5 µM 14-3-3-LgBiT RC, 1 µM VF-Sensor, 50 nM RSK2, 40 µM coelenterazine (#301-10 hCTZ, Prolume Ltd., Pinetop, AZ, USA), 1 mM DTT, 6 mg/mL BSA in 1X kinase buffer (50 mM HEPES, pH = 7.4; 100 mM NaCl; 5 mM MgCl2; 5% glycerol; and 0.05% Igepal). In the case of the MAPK-PhALC assays the reaction mixture contained 1 µM RC (WW-LgBiT RC), 1 µM MAPK-Sensor (SmBiT-constructs), 0.3–2 nM double-phosphorylated MAPKs, and 60 µM coelenterazine in 1X kinase buffer. Measurements were performed using a BioTek Synergy 2 or Cytation 3-plate reader at 25 °C in white 96- or 384-well plates (SPL Life Sciences, Gyeonggi-do, Korea, #30196 or Greiner Microplate, Greiner Bio-One AG, Kremsmünster, Austria, #781904). The reaction was induced by the addition of ATP (at 0.1 or 1 mM final concentration) dissolved in 1X kinase buffer. Peptide inhibitor stocks were at 10 mM concentration in buffer (50 mM Tris, pH = 8.0), while the stocks of the other inhibitors were at 10 mM in DMSO. The luminescence signal was monitored for 30–60 min and the slope from the initial linear region was calculated by linear regression. For IC_50_ determination selected inhibitors or peptides were serial diluted in the indicated range. Luminescence signal was observed for a duration of up to 30 min. During this time, we specifically looked at the initial linear phase, which usually occurred within the first 5 min. To quantify the rate of change in the signal during this early period, we used linear regression analysis to calculate the slope. The initial slopes obtained in this manner were normalized using the positive and negative controls, and then a dose-response curve was fitted to the obtained values in Origin 2018 (DoseResponse function).

ATP-competitive inhibitors were purchased: SL0101 from MedChemExpress (Monmouth Junction, NJ, USA), #HY-15237; staurosporin from SelleckChem (Houston, TX, USA), #S1421; SCH772984 from SelleckChem, #S7101; SB202190 from SelleckChem, #S1077; JNK-IN-8 from SelleckChem, #S4901,> and SP600125 from SelleckChem, #S1460. The docking peptides added in trans into the PhALC reaction mix were chemically synthesized (pepMNK1: MKLSPPSKSRLARRRALA, pepNFAT4: LERPSRDHLYLPLE, and pepFENEF: GLFNELASPFENEFKKAS) using Fmoc/^t^Bu chemistry. ORF45_17mer (PTVIDMSAPDDVFAEDT) was purchased from NovoPro Bioscience Inc. (Shanghai, China).

### 4.6. Academic-Compound Collections and Screening

The in-house academic compound library of the Research Center for Natural Sciences, Budapest, was assembled from chemist groups comprised of mostly small, fragment-like compounds (~500). 88 compounds from this were chosen based on Tanimoto index similarity analysis using KNIME to represent the full diversity of the larger compound collection in a smaller set [57,58]. The reaction mix for academic compound screening was 50 mM HEPES, pH = 7.4; 100 mM NaCl; 5 mM MgCl_2;_ 5% glycerol; and 0.05% Igepal, which included a kinase at concentrations ranging from 0.5 to 10 nM, two luciferase constructs at 1-1 μM, and coelenterazine at a concentration of 60 μM. In the case of RSK measurements, the solution also contained 6 mg of BSA. Inhibitors were added to achieve a final concentration of 200 μM using the Mosquito pipetting robot. The reactions were initiated by adding 15 μL of an ATP-containing solution, resulting in a final ATP concentration of 100 μM in the reaction mix, and the reaction was monitored for 10 min. Subsequently, a linear fit was applied to the obtained luminescent signal data using the NumPy library from Python. The Z-score calculations were done using the following formula: Z = 1 − [3 × (σ_p_ + σ_n_)/|μ_p_ − μ_n_|], where σ_p_ corresponds to the SD of positive control wells and σ_n_ to negative controls or to INH wells (containing 200 μM docking inhibitors in trans), while μ_p_ and μ_n_ are the averages (calculated from 6 wells from two plates).

## Data Availability

The bacterial expression plasmid DNA constructs for VF-Sensor, MEF2A-Sensor, PDE4B_Sensor, FENEF-Sensor, RC(WW)-LgBit, and RC(14-3-3ε)-LgBit are deposited into the Addgene plasmid database with the following ID numbers: 208927, 208928, 208929, 208930, 208926, and 208931, respectively.

## Supplementary Materials

The following supporting information can be downloaded at: https://www.mdpi.com/article/10.3390/ijms241914854/s1.
