# Supplementary material for: Phosphorylation-Assisted Luciferase Complementation Assay Designed to Monitor Kinase Activity and Kinase-Domain-Mediated Protein–Protein Binding
Source: Int J Mol Sci. 2023 Oct 3;24(19):14854. doi: 10.3390/ijms241914854 (PMC10573712; doi:10.3390/ijms241914854)
Supplement: Supplementary file 1 [file ijms-24-14854-s001.zip › ijms-2638960-supplementary.pdf]

Supplementary Material

Phosphorylation-assisted Luciferase Complementation assay designed to monitor kinase activity and kinase domain mediated protein-protein binding

Ádám L. Póti, Laura Dénes, Kinga Papp, Csaba Bató, Zoltán Bánóczi, Attila Reményi, Anita Alexa

Figure S1. Results of screening with an academic compound collection
Summary of five PhALC assay screens with an academic compound collection (RIH compounds: 88 molecules). Red bars show the positive control initial rates (no compound added) while negative control indicates reactions without enzyme or INH corresponds to measurements where a known docking peptide or an inhibitor was added *in trans* in a concentration (200 µM) that eliminated docking *in cis*. Error bars indicate SD based on two experiments (N=2). The 96-well plates had the same layout regarding the experimental 88 compounds.

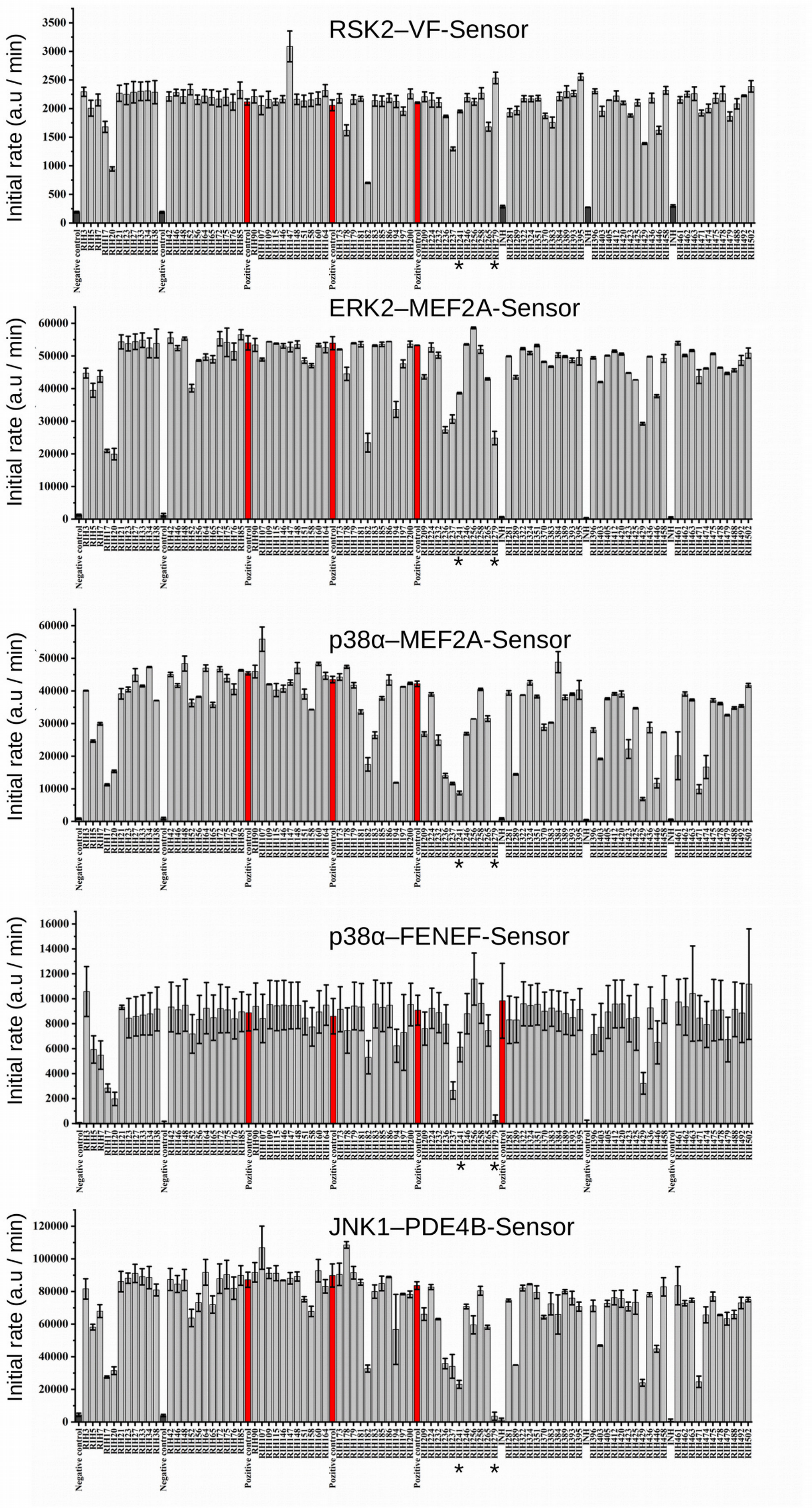
RSK2–VF-Sensor
ERK2–MEF2A-Sensor
p38α–MEF2A-Sensor
p38α–FENEF-Sensor
JNK1–PDE4B-Sensor
Initial rate (a.u / min)
Negative control
Pozitive control
*
